# Five-year cost-effectiveness analysis of the European Fans in Training (EuroFIT) physical activity intervention for men versus no intervention

**DOI:** 10.1186/s12966-020-00934-7

**Published:** 2020-03-04

**Authors:** Spyros Kolovos, Aureliano P. Finch, Hidde P. van der Ploeg, Femke van Nassau, Hana M. Broulikova, Agni Baka, Shaun Treweek, Cindy M. Gray, Judith G. M. Jelsma, Christopher Bunn, Glyn C. Roberts, Marlene N. Silva, Jason M. R. Gill, Øystein Røynesdal, Willem van Mechelen, Eivind Andersen, Kate Hunt, Sally Wyke, Judith E. Bosmans

**Affiliations:** 1 Department of Health Sciences, Faculty of Science, Vrije Universiteit Amsterdam, Amsterdam Public Health research institute, Amsterdam, The Netherlands; 2 Nuffield Department of Orthopaedics, Rheumatology and Musculoskeletal Sciences, University of Oxford, Oxford, UK; 3 Amsterdam UMC, VU medical center, Department of Public and Occupational Health, Amsterdam Public Health research institute, Van der Boechorststraat 7, NL-1081 BT Amsterdam, The Netherlands; 4 Health Services Research Unit, University of Aberdeen, Aberdeen, UK; 5 Institute of Health and Wellbeing, College of Social Sciences, University of Glasgow, Glasgow, UK; 6 Department of coaching and psychology, Norwegian School of Sport Science, Oslo, Norway; 7 CIPER, Faculdade de Motricidade Humana, Universidade de Lisboa, Lisbon, Portugal; 8 Faculdade de Educação Física e Desporto, Universidade Lusófona de Humanidades e Tecnologias, Lisbon, Portugal; 9 Institute of Cardiovascular and Medical Sciences, College of Medical, Veterinary and Life Sciences, University of Glasgow, Glasgow, UK; 10 Department of Teacher Education, NLA University College, Bergen, Norway; 11 Institute for Social Marketing and Health, University of Stirling, Stirling, UK

## Abstract

**Objectives:**

Increasing physical activity reduces the risk of chronic illness including Type 2 diabetes, cardiovascular disease and certain types of cancer. Lifestyle interventions can increase physical activity but few successfully engage men. This study aims to investigate the 5 year cost-effectiveness of EuroFIT, a program to improve physical activity tailored specifically for male football (soccer) fans compared to a no intervention comparison group.

**Methods:**

We developed a Markov cohort model in which the impact of improving physical activity on five chronic health conditions (colorectal cancer, Type 2 diabetes, coronary heart disease, stroke and depression) and mortality was modelled. We estimated costs from a societal perspective and expressed benefits as quality adjusted life years (QALYs). We obtained data from a 4-country (England, Netherlands, Portugal and Norway) pragmatic randomised controlled trial evaluating EuroFIT, epidemiological and cohort studies, and meta-analyses. We performed deterministic and probabilistic sensitivity analyses to assess the impact of uncertainty in the model’s parameter values on the cost-effectiveness results. We used Monte Carlo simulations to estimate uncertainty and presented this using cost-effectiveness acceptability curves (CEACs). We tested the robustness of the base case analysis using five scenario analyses.

**Results:**

Average costs over 5 years per person receiving EuroFIT were €14,663 and per person receiving no intervention €14,598. Mean QALYs over 5 years were 4.05 per person for EuroFIT and 4.04 for no intervention. Thus, the average incremental cost per person receiving EuroFIT was €65 compared to no intervention, while the average QALY gain was 0.01. This resulted in an ICER of €5206 per QALY gained. CEACs show that the probability of EuroFIT being cost-effective compared to no intervention is 0.53, 0.56 and 0.58 at thresholds of €10,000, €22,000 and €34,000 per QALY gained, respectively. When using a time horizon of 10 years, the results suggest that EuroFIT is more effective and less expensive compared to (i.e. dominant over) no intervention with a probability of cost-effectiveness of 0.63 at a threshold of €22,000 per QALY gained.

**Conclusions:**

We conclude the EuroFIT intervention is not cost-effective compared to no intervention over a period of 5 years from a societal perspective, but is more effective and less expensive (i.e. dominant) after 10 years. We thus suggest that EuroFIT can potentially improve public health in a cost-effective manner in the long term.

## Introduction

Physical activity decreases the risk of non-communicable diseases such as coronary heart disease, Type 2 diabetes, colorectal cancer, stroke and depression [1–4]. Some benefits from physical activity occur quickly, such as reduced blood pressure, and improved sleep, cognitive function and insulin sensitivity; others, such as increased cardiorespiratory fitness, decreased depressive symptoms, and sustained reduction in blood pressure, only accrue over months or years of increased physical activity [1]. International guidelines demonstrate that substantial health improvements can be achieved by performing moderate to vigorous physical activity at least 150 min per week [5], which equates to at least 450 Metabolic Equivalent of Task minutes (MET-min) per week [6]. Although estimates vary, many adults do not meet these guidelines recommendations [7, 8], resulting in substantial potentially preventable morbidity and mortality [2, 5, 9, 10], as well as high societal and healthcare costs [11].

Increasing physical activity reduces the risk of chronic illness including Type 2 diabetes, cardiovascular disease and certain types of cancer [12–14]. Lifestyle interventions can increase physical activity but few successfully engage men. In response to this, Gray et al. developed a program, Football Fans in Training (FFIT), tailored specifically to men. FFIT aimed to engage and support men to lose weight through dietary change and physical activity by working with predominant constructions of masculinity [15–17]. Based on the results of a pragmatic randomised controlled trial (RCT), FFIT participants lost more weight over 12 months than control participants (mean difference in percentage weight loss 4.36% (95% confidence interval (CI) 3.64 to 5.08) [17]. Moreover, weight loss was sustained over 3.5 years of follow-up (mean percentage weight loss from baseline 2.36% (95% CI 1.41 to 3.31) [18].

Recently, the European Fans in Training (EuroFIT) lifestyle change program was developed. The EuroFIT program built on the FFIT program, but shifted the focus of the program from weight loss to improving physical activity and reducing sedentary behaviour. EuroFIT was rigorously evaluated in a 4-country RCT [19]. While the EuroFIT program successfully increased the number of daily steps at 12 months, there was no difference in sedentary time between the two groups [20]. However, improvements were observed in secondary outcomes, including body weight, the proportion of participants with a BMI less than 30 kg/m^2^, waist circumference, well-being, self-esteem and vitality, and biomarkers of cardiovascular health (i.e. systolic and diastolic blood pressure, fasting insulin and fasting triglycerides). The within-trial cost-effectiveness analysis showed that EuroFIT was not cost-effective compared to a waitlist condition at 12 months follow-up for quality adjusted life years (QALYs) [20]. However, if the observed improvements in physical activity are sustained over time, it is possible that EuroFIT is cost-effective with regard to QALYs in the long term. Because the waiting list comparison group received the EuroFIT program after conclusion of the RCT, longer follow-up within the RCT was unfeasible. Therefore, we used a Markov cohort model to estimate the five-year cost-effectiveness of the EuroFIT program compared to no intervention. This is one of the first longer-term cost-effectiveness studies evaluating a physical activity program specifically tailored to men.

## Methods

### Design

We developed a Markov cohort model with a time horizon of 5 years to estimate the cost-effectiveness of the EuroFIT program from a societal perspective [19]. We chose to use a Markov model due to its flexibility and its ability to handle multiple possible outcomes [21]. We estimated transition probabilities, costs and utilities using data from both the previously conducted EuroFIT RCT and the literature.

### The EuroFIT RCT

The EuroFIT RCT was conducted during 2016 and 2017 in England, The Netherlands, Norway and Portugal. Ethics committees in each of the four countries have approved the study protocol [19]. The RCT was registered with ISRCTN number 81935608.

Details of the EuroFIT RCT can be found in Van Nassau et al. [19] and Wyke et al. [20]. In summary, 15 professional football clubs in England, the Netherlands, Norway, and Portugal recruited 1113 men aged 30–65 with self-reported body mass index (BMI) ≥27 kg/m2 into the trial using any of social media posting, email invitations to club members, and local press coverage. We describe baseline characteristics of the participating men in Supplementary Table 1.

### Interventions

We designed the EuroFIT program to support men in becoming more physically active. A detailed description of the EuroFIT intervention is available in Van Nassau et al. [19] and Van de Glind et al. [22]. Briefly, the EuroFIT intervention consisted of 12 weekly sessions in groups of 15 to 20 men. Sessions lasted for 90 min, and combined classroom discussions with group-based physical activities tailored to the ability of the participants. Club coaches were trained to create a positive motivational climate tailored specifically to men while delivering the intervention. Coaches also taught participants to choose from a ‘toolbox’ of behavior change techniques, and emphasized personally-relevant benefits of behavior change such as being better able to fulfil valued activities and roles.

The comparison group in the RCT was on a waiting list for the 12 months of the RCT. Subsequently, participants in the comparison group were offered the EuroFIT intervention. However, for the current paper we assumed that they received no intervention during the time horizon of the model. Thus, the control group constitutes a no intervention comparison group.

### Model structure and population

**Fig. 1:**
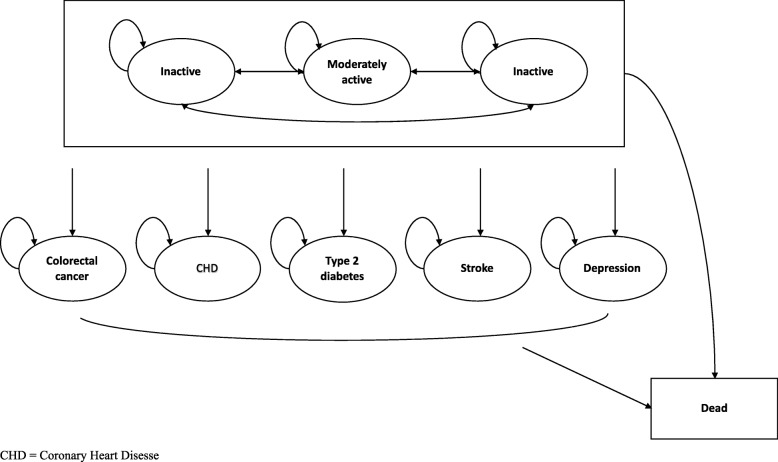
Structure of the Markov model. CHD = Coronary Heart Disease

We implemented the Markov model in R software. We based the model on previously published physical activity models [23–27] and extended it with a depression health state, as there is evidence of depression being associated with lack of physical activity [28, 29]. The Markov model comprised nine mutually exclusive health states. Three health states described the activity levels: physically inactive, moderately active and recommended level of physical activity. Five health states covered five health conditions associated with a lack of physical activity: colorectal cancer, coronary heart diseases, stroke, Type 2 diabetes and depression. The absorbing ninth state was death. Figure 1 shows the structure of the Markov model.

The target population was the same as the participants of the EuroFIT RCT. Apart from having a BMI of 27 or more, we assumed the population otherwise to be in good health, which was defined as the absence of any of the five health conditions included in the model. We determined the proportion of participants starting in each activity level based on the proportion of participants meeting inactive (17.5%), moderately active (10.5%) and recommended activity (72.0%) thresholds at baseline in the EuroFIT RCT. At the end of each cycle, participants could remain in their assigned physical activity category, or move to a different category of physical activity, a health condition or death. The cycle length of the model was 1 year. Thus, we assumed that transitions between the different states of the Markov model occurred yearly. We calculated expected costs and QALYs over a time span of 5 years. In the base case analysis, we assumed that the beneficial effect of EuroFIT compared to no intervention on physical activity over 12 months remained stable for the 5 years of the model. We tested this assumption in one of the sensitivity analyses.

### Model input parameters

Table 1 presents the parameters used in the Markov model. As a first line strategy to retrieve information on the association between physical activity levels and individuals’ health, we used the 2018 Physical Activity Guidelines Advisory Committee Scientific Report [6]. The report includes the broadest systematic literature review available on the association between physical activity and adverse outcomes, including risk of cardiovascular disease, cancers and all-cause mortality. As it does not specifically focus on the population investigated in EuroFIT, we used this review to identify studies including male individuals aged between 30 to 65 years.

**Table 1:** Model input parameters

PARAMETERS FOR THE BASE CASE ANALYSIS			
	Values*	Distribution for probabilistic sensitivity analysis	Source
**Physical activity (transition probabilities at the end of each cycle of 12 months)**			
*EuroFIT*			
Inactive to Inactive	0.18	Beta (α = 4.0, β = 17.0)	EuroFIT RCT [20]
Inactive to Moderately active	0.054	Beta (α = 0.34, β = 5.7)	EuroFIT RCT [20]
Inactive to Recommended activity	0.71	Beta (α = 59.4, β = 19.6)	EuroFIT RCT [20]
Moderately active to Moderately active	0.042	Beta (α = 0.09, β = 1.9)	EuroFIT RCT [20]
Moderately active to Inactive	0.085	Beta (α = 0.44, β = 4.56)	EuroFIT RCT [20]
Moderately active to Recommended activity	0.82	Beta (α = 33.8, β = 5.2)	EuroFIT RCT [20]
Recommended activity to Recommended Activity	0.89	Beta (α = 357.9, β = 26.1)	EuroFIT RCT [20]
Recommended activity to Inactive	0.042	Beta (α = 0.83, β = 18.2)	EuroFIT RCT [20]
Recommended activity to Moderately active	0.023	Beta (α = 0.24, β = 9.8)	EuroFIT RCT [20]
*No intervention*			
Inactive to Inactive	0.33	Beta (α = 11.5, β = 21.5)	EuroFIT RCT [20]
Inactive to Moderately active	0.12	Beta (α = 1.6, β = 10.4)	EuroFIT RCT [20]
Inactive to Recommended activity	0.49	Beta (α = 25.0, β = 23.0)	EuroFIT RCT [20]
Moderately active to Moderately active	0.17	Beta (α = 1.5, β = 6.6)	EuroFIT RCT [20]
Moderately active to Inactive	0.19	Beta (α = 2.1, β = 8.0)	EuroFIT RCT [20]
Moderately active to Recommended activity	0.58	Beta (α = 16.6, β = 10.4)	EuroFIT RCT [20]
Recommended activity to Recommended Activity	0.80	Beta (α = 290.7, β = 58.3)	EuroFIT RCT [20]
Recommended activity to Inactive	0.10	Beta (α = 4.2, β = 37.8)	EuroFIT RCT [20]
Recommended activity to Moderately active	0.064	Beta (α = 1.9, β = 26.1)	EuroFIT RCT [20]
**Health conditions (transition probabilities at the end of each cycle of 12 months)**			
Inactive to colorectal cancer	0.015	Fixed	COSM study [30, 31]
Inactive to heart diseases	0.011	Fixed	Meta-analysis; SALLS study [30, 32–34]
Inactive to Type 2 diabetes	0.005	Fixed	EPIC-Interact study and epidemiological study [33, 35, 36]
Inactive to stroke	0.0046	Fixed	ARIC study [37]
Inactive to depression	0.010	Fixed	Meta-analysis; Health survey for England and Scotland [34, 35, 38]
Moderately active to colorectal cancer	0.011	Fixed	COSM study [30, 31]
Moderately active to heart disease	0.009	Fixed	Meta-analysis; SALLS study [30, 32–34]
Moderately active to Type 2 diabetes	0.0038	Fixed	EPIC-Interact study; epidemiological study [33, 35, 36]
Moderately active to stroke	0.0033	Fixed	ARIC study [37]
Moderately active to depression	0.0094	Fixed	Meta-analysis; Health survey for England and Scotland [34, 35, 38]
Recommended activity to colorectal cancer	0.0096	Fixed	COSM study [30, 31]
Recommended activity to heart disease	0.008	Fixed	Meta-analysis; SALLS study [30, 32–34]
Recommended activity to Type 2 diabetes	0.0033	Fixed	EPIC-Interact study; epidemiological study [33, 35, 36]
Recommended activity to stroke	0.0029	Fixed	ARIC study [37]
Recommended activity to depression	0.0092	Fixed	Meta-analysis; Health survey for England and Scotland [34, 38, 39]
Mortality (transition probabilities at the end of each cycle of 12 months)			
Inactive to death	0.016	Fixed	Meta-analysis; epidemiological study [73, 74, 75, 76]
Moderately active to death	0.012	Fixed	Meta-analysis; epidemiological study [73, 74, 75, 76]
Recommended activity to death	0.010	Fixed	Meta-analysis; epidemiological study [73, 74, 75, 76]
Colorectal cancer to death	0.092	Fixed	International Cancer Benchmarking partnership registries [40]
Coronary heart disease to death	0.002	Fixed	WONDER registry [41]
Type 2 diabetes to death	0.015	Fixed	ZODIAC study [42]
Stroke to death	0.400	Fixed	MONICA registry [43]
Depression to death	0.030	Fixed	STIRLING registry [44]
Utility values			
Inactive – Base case	0.909	Beta (α = 3.0, β = 0.38)	EuroFIT RCT [20]
Moderately active – Base case	0.919	Beta (α = 5.1, β = 0.51)	EuroFIT RCT [20]
Recommended activity – Base case	0.922	Beta (α = 5.1, β = 0.43)	EuroFIT RCT [20]
Colorectal cancer	0.786	Fixed	Systematic review [45]
Coronary hearth disease	0.735	Fixed	Longitudinal survey [46]
Stroke	0.62	Fixed	Longitudinal survey [47]
Type 2 diabetes	0.785	Fixed	Systematic review [48]
Depression	0.57	Fixed	Systematic review [49]
**Annual costs per person according to the societal perspective (€ 2017)**			
Inactive	2436	Gamma (shape = 0.19, scale = 12,658)	EuroFIT RCT [20]
Moderately active	1506	Gamma (shape = 0.22, scale = 6920)	EuroFIT RCT [20]
Recommended activity	1997	Gamma (shape = 0.24, scale = 8222)	EuroFIT RCT [20]
Colorectal cancer	34,085	Fixed	Cross sectional study; Health insurance registry [50, 51]
Coronary heart disease	5239	Fixed	Economic burden [52]
Type 2 diabetes	5907	Fixed	Economic burden [53]
Stroke	24,979	Fixed	Economic burden [54]
Depression	6819	Fixed	Cost-effectiveness analysis [55]
EuroFIT program	260	Fixed	EuroFIT RCT [20]
**PARAMETERS FOR THE SENSITIVITY ANALYSES**			
Utility values			
Inactive – Literature utilities	0.80	Fixed	Economic evaluation [24]
Moderately active – Literature utilities	0.87	Fixed	Economic evaluation [24]
Recommended activity – Literature utilities	0.91	Fixed	Economic evaluation [24]
**Annual costs per person according to the healthcare perspective (€ 2017)**			
Inactive	1107	Gamma (shape = 0.10, scale = 10,924)	EuroFIT RCT [20]
Moderately active	594	Gamma (shape = 0.35, scale = 1707)	EuroFIT RCT [20]
Recommended activity	747	Gamma (shape = 0.19, scale = 4040)	EuroFIT RCT [20]
Colorectal cancer	25,346	Fixed	Cross sectional study; Health insurance registry [50, 51]
Coronary heart disease	1954	Fixed	Economic burden [52]
Type 2 diabetes	3089	Fixed	Economic burden [53]
Stroke	18,750	Fixed	Economic burden [54]
Depression	966	Fixed	Cost-effectiveness analysis [55]

*Values represent probabilities, mean utilities or mean costs

To identify additional studies on the natural course of physical activity, we conducted targeted literature searches using PubMed, Web of Science and Google Scholar. We only included studies written in English. Whenever a study provided data that were relevant to populate the model, we screened the reference list to identify and retrieve additional evidence. We also conducted targeted literature searches to retrieve information on resource use and utilities associated with physical activity levels. Whenever possible, we retrieved evidence from studies conducted in European countries related to a mainly Caucasian, male, overweight population, aged between 30 and 65 years old. If these were not available, we used comparable evidence from normal weight individuals, from the USA, or for male population of an older age as model inputs.

### Physical activity

Table 1 presents the changes in self-reported physical activity for the EuroFIT and no intervention groups. We estimated yearly probabilities of transitioning between physical activity levels or staying in the original activity level using the EuroFIT pragmatic RCT [20] and made them conditional on the transition probabilities of progressing to a condition or death. The EuroFIT RCT assessed physical activity both objectively with the activPAL monitor (model activPALTM micro; PAL Technologies Ltd., Glasgow, UK) and subjectively using the self-reported International Physical Activity Questionnaire (IPAQ short form), which assesses walking, other moderate intensity physical activity and vigorous intensity physical activity [56]. Both measures of physical activity improved in the EuroFIT arm of the RCT. We used self-reported physical activity in our model for two main reasons. First, although subjectively reported physical activity rates almost certainly over-estimate the actual level of physical activity, the current physical activity guidelines are primarily built on epidemiologic studies that used self-report measures. Secondly, there are still too few studies that estimate the association between objective measures of physical activity and health.

We defined the three activity categories according to the self-reported MET-min per week of the EuroFIT participants. MET-min per week is an index capturing the total amount of all physical activity, where one MET is defined as the rate of energy expenditure at rest [5]. We classified participants reporting < 200 MET-min per week as physically inactive, participants reporting between 200 and 450 MET-min per week as moderately physically active, and participants reporting > 450 MET-min per week as meeting desired levels of physical activity, according to physical activity guidelines [5, 6, 57]. We estimated the probability of moving to a higher or lower physical activity category in the EuroFIT RCT for the intervention and the comparison group separately as the percentage of participants in one activity group at baseline moving to a different activity group at 12 months follow up.

### Health conditions and mortality

To estimate the yearly probability of developing each of the five health conditions included in the model, we used incidence and relative risk data from the literature. To estimate the probability of death associated with the five conditions, we used annual mortality rates based on a particular condition. To estimate the probability of death associated with different levels of physical activity, we used mortality rates and relative risk data. The sources are described in more detail in Table 1; they included meta-analyses, epidemiological studies and registries [30–33, 35–44, 55, 57–60].

### Utilities

We used QALYs as the effect measure in the cost-effectiveness analysis. QALYs are calculated by multiplying the time participants spend in a given health state with a utility value that represents the health-related quality of life (HRQoL) associated with that health state. We obtained utility values for the inactive, moderately active and recommended activity states from the EuroFIT RCT [20]. The EuroFIT RCT used the five level version of the EuroQol questionnaire (EQ-5D) to estimate utilities, which is the most widely used measure to do this [61]. We obtained utilities for the chronic conditions covered in the model from three systematic reviews and two longitudinal studies [45–49].

### Costs

We estimated costs of the EuroFIT program using a bottom-up approach, and included costs of personnel responsible for preparation, coordination, administration, recruitment and program delivery, and materials. We estimated costs generated in the different activity states based on the EuroFIT RCT [20]. We obtained costs of the conditions modelled from three economic burden studies, one cross-sectional study, one registry study and one economic evaluation [50–55]. In accordance with the societal perspective employed in this study, we included both healthcare costs and lost productivity costs. We adjusted costs using the consumer price index to the year 2017 [62] and, whenever needed, we converted costs into Euros (€) for the year 2017 using purchasing power parities [63], as recommended in international guidelines [21].

### Cost-effectiveness analysis

We distributed a cohort of 10,000 participants in each of the treatment arms over the three activity categories at the start of the time horizon modelled. As recommended by the National Institute for Health and Care Excellence (NICE), we discounted costs and effects at 3.5% [64]. We calculated incremental cost-effectiveness ratios (ICERs) by dividing the difference in total costs between the EuroFIT intervention and the comparison group by the difference in total QALYs. We consider the intervention cost-effective if the ICER is smaller than a pre-defined willingness-to-pay threshold. For the current study, we used the commonly accepted NICE threshold of £20,000 to £30,000 per QALY gained [64], which corresponds to €22,000 to €34,000 per QALY gained.

### Sensitivity analyses

#### Probabilistic sensitivity analyses

We performed a probabilistic sensitivity analysis (PSA) for the base case analysis and each of the four scenario analyses. For parameters that were directly observed in the EuroFIT RCT, we estimated a sampling distribution based on the variance around the point estimates for these parameters. Next, we used Monte Carlo simulation (25,000 simulations) to randomly select values from the specified distributions. We used beta distributions for transition probabilities, beta distributions for utilities and gamma distributions for costs (Table 1). Using the 25,000 simulations, we estimated 95% credibility intervals around incremental costs and QALYs based on the 2.5 and 97.5% percentiles. Finally, we estimated cost-effectiveness acceptability curves (CEACs) to present the probability of EuroFIT being cost-effective compared to no intervention at different willingness to pay thresholds.

#### Deterministic sensitivity analyses

We performed deterministic sensitivity analyses in which the discount rates for costs and effects were varied (0 and 5%). This was undertaken for costs and effects separately, and for costs and effects simultaneously.

#### Scenario analyses

We tested the robustness of the base case findings by performing four scenario analyses. For all scenarios, we evaluated the deterministic impact on the ICER point estimate and the probabilistic impact on the probability of cost-effectiveness at different willingness to pay ratios.

First, we extended the time horizon of the model to 10 years to assess longer term effects of the intervention while assuming that the effect of EuroFIT was maintained over a period of 10 years. Second, we ran the model from the healthcare perspective, as this is preferred by health technology assessment bodies such as NICE [64]. Third, we obtained utility values for the inactive, moderately active and recommended activity states from the literature, and specifically from a previously conducted economic evaluation of an intervention to improve physical activity [24]. We did this sensitivity analysis, because differences in utility values between physical activity levels in the literature are larger than the ones we found in the EuroFIT RCT. Fourth, we re-ran the model while limiting the beneficial effect of EuroFIT on physical activity to the first year, hence after the first year the transition probabilities from the no intervention group were used.

## Results

### Cost-effectiveness analysis

Table 2 reports the results of the base-case analysis. Total costs for the EuroFIT group were €146,629,613 per 10,000 participants as compared to €145,975,002 in the no intervention group. This resulted in an incremental cost for the cohort of €654,611, equivalent to €65 per participant. QALYs in the EuroFIT group were 40,431 compared to 40,405 in the no intervention group, resulting in a small gain of 126 QALYs (+ 0.31%) in favour of EuroFIT, which is equivalent to 0.013 QALYs gained per participant. EuroFIT generates 195 QALYs more than no intervention based on time spent in the health states describing the physical activity levels (the ‘healthy’ states). In addition, the EuroFIT group generates 69 QALYs less than no intervention based on time spent in the health states describing the associated health conditions (the ‘disease’ states). The resulting ICER was €5206 per QALY gained for EuroFIT in comparison with no intervention.

**Table 2:** Cost effectiveness results for the base case and scenario analyses

Analysis	Treatment arm	Total costs (€ 2017)	Total QALYs	Incremental Cost	Incremental QALYs	ICER
Base case	EuroFIT	€146,629,613	40,531	€654,611 (−73,893,166; 81,741,624)	126 (−1999; 2527)	5206
	No intervention	€145,975,002	40,405			
Healthcare perspective	EuroFIT	€72,489,139	40,531	€496,731 (−37,026,528; 35,107,767)	126 (− 2026; 2537)	3951
	No intervention	€71,992,408	40,405			
Utility values from the literature	EuroFIT	€146,629,613	39,767	€654,611 (−73,893,166; 81,741,624)	564 (−68; 1106)	1161
	No intervention	€145,975,002	39,203			
EuroFIT effectiveness lasts only 1 year	EuroFIT	€147,631,100	40,427	€1,759,289 (−19,869,112; 25,189,547)	52 (− 555; 725)	33,997
	No intervention	€145,871,811	40,375			
Time horizon 10 years	EuroFIT	€303,622,814	71,010	-€2,595,287 (−128,814,119; 137,269,728)	373 (− 3254; 4534)	Dominant
	No intervention	€306,218,101	70,636			

*QALY* Quality-Adjusted Life-Year, *ICER* Incremental Cost-Effectiveness Ratio

#### Probabilistic sensitivity analysis

**Fig. 2:**
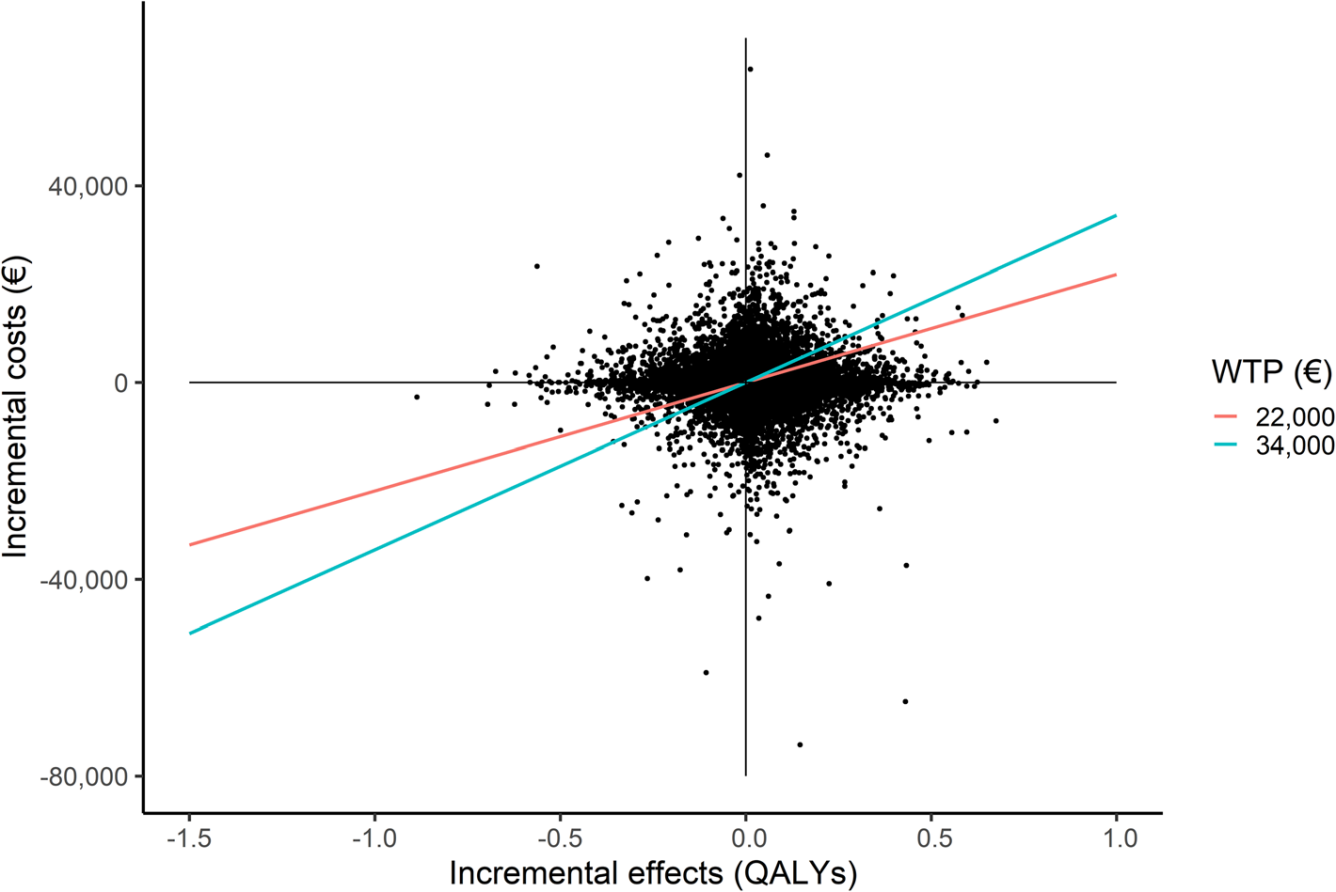
Cost-effectiveness plane for the base case analysis. WTP = Willingness-To-Pay; QALY = Quality-Adjusted Life-Year

Figure 2 presents the distribution of incremental cost-effect pairs for the base case analysis based on the 25,000 simulations in the probabilistic sensitivity analysis. The cost-effectiveness plane shows that EuroFIT is more effective and more costly than no intervention, and that there is considerable uncertainty around the ICER.

**Fig. 3:**
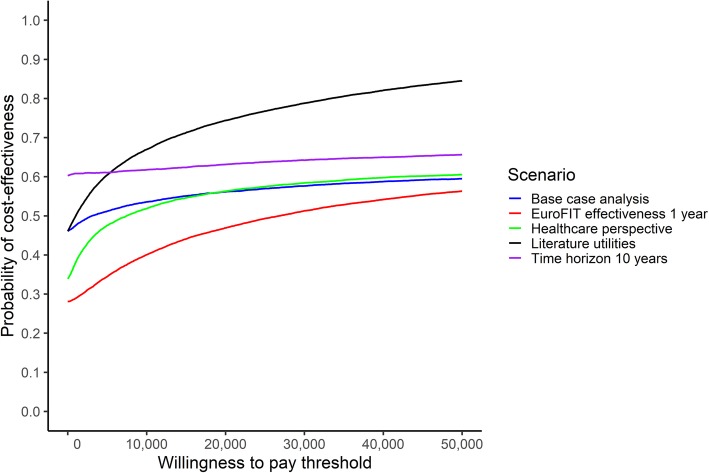
Cost-effectiveness acceptability curves for the base case analysis and the scenario analyses

From the CEAC (Fig. 3), it emerges that the probability that EuroFIT is cost-effective compared with no intervention, is 0.53 at a threshold of €10,000 per QALY. This probability increases to 0.56 and 0.58 at thresholds of €22,000 and €34,000 per QALY gained, respectively.

### Sensitivity analyses

#### Deterministic sensitivity analyses

**Fig. 4:**
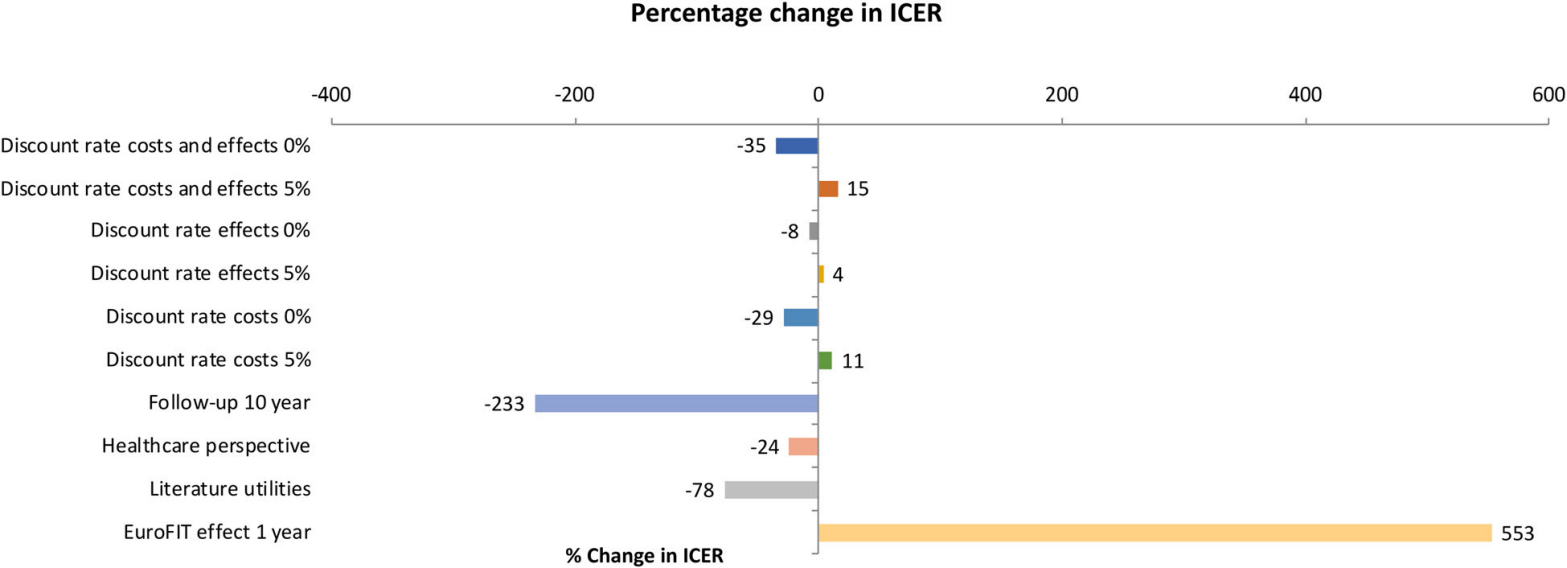
Tornado diagram showing the change in incremental cost-effectiveness ratios between the base case analysis and the scenario and deterministic sensitivity analyses

Figure 4 reports the results for the deterministic sensitivity analyses in which the discount rates were varied from the base case analysis. The figure shows that the effect on the ICER point estimates was limited with impacts ranging from − 35% (discount rate for costs and effects 0%) to 15% (discount rate for costs and effects 5%).

#### Scenario analyses

Figure 4 also presents the deterministic results of the scenario analyses. When the time horizon of the model was extended to 10 years, the ICER became negative due to a negative difference in costs (−€2,595,287) and a positive difference in QALYs gained (373 QALYs gained), indicating that EuroFIT is dominant over no intervention. Employing a healthcare perspective decreased the ICER by 24%. Using literature estimates for the utilities associated with the different physical activity levels resulted in 39,767 and 39,203 QALYs in the EuroFIT and no intervention group, respectively. Thus, the total number of QALYs gained was 564 in the EuroFIT group, or 0.056 QALY per participant. The ICER was €1161 per QALY gained, indicating a decrease of 78% compared with the base case analysis. The assumption that the effect of EuroFIT lasted only for 1 year, resulted in an increase of the difference in costs between EuroFIT and no intervention (€1,759,289) and a decrease in QALYs gained (52 QALYs gained). As a result, the ICER increased by 553% from €5206 to €33,996 per QALY gained. Although there was a considerable impact on the point estimate of the ICER for all scenarios considered, the probability of EuroFIT being cost-effective compared with no intervention changed less. When assuming one-year effects of EuroFIT, the probability was 0.47 at a threshold of €22,000 per QALY gained, when using a healthcare perspective the probability was 0.57, and when modelling a time horizon of 10 years the probability was 0.63 (Fig. 3). In the scenario using literature utilities, the probability that EuroFIT is cost-effective compared to no intervention was 0.75 at a threshold of €22,000 per QALY gained (Fig. 3).

## Discussion

In this study, we evaluated the long-term cost-effectiveness of EuroFIT compared to no intervention from a societal perspective. EuroFIT was slightly more effective and slightly more expensive than no intervention, resulting in an ICER of €5206 per QALY gained. Although this point estimate of the ICER lies well below the commonly accepted thresholds of between €22,000 to €34,000 per QALY gained, the probability of cost-effectiveness at these thresholds was only 56 to 58%. Using a 10-year time horizon and assuming 1 year effects of EuroFIT had the largest impact on the ICER point estimates. However, the change in the probability of cost-effectiveness was largest in the scenario where utility values from the literature were used for the three physical activity levels. Given this evidence, we consider the EuroFIT intervention not to be cost-effective compared to no intervention on a time horizon of 5 years, but cost-effective on a time horizon of 10 years.

When interpreting the findings of the current study, it is important to consider the fact that at baseline already 72% of the participants were at recommended levels of activity. Since we modelled only effects on physical activity in the current study, this leaves little room for improvement. However, EuroFIT resulted in considerable improvements in other outcomes, such as weight and biomarkers of cardiometabolic health, as well [20]. As these improvements might also positively impact health benefits, it is likely that our study underestimates the cost-effectiveness of the intervention.

The utility values derived from the EuroFIT RCT were similar for the three levels of physical activity. In addition, utility values reported by the EuroFIT RCT participants in all three physical activity states were around 0.90, suggesting that the participants had high quality of life already. This is in contrast with utility values reported in previous studies, which have shown that the individuals following the recommended guidelines for physical activity have increased utility values [65–67]. This might reflect problems with the construct validity of the EQ-5D-5 L in the population investigated. That is, the EQ-5D-5 L might miss important dimensions relevant to assess health benefits resulting from behavioural interventions, examples of which can be energy, vitality, enthusiasm, sleeping, relationships and satisfaction. Using multi-attribute approaches that include such domains, as suggested by Wildman and Wildman may prove to be helpful when evaluating complex interventions like EuroFIT [65, 68].

Our findings are in line with previous studies evaluating the cost-effectiveness of intervention programs targeted to improve physical activity. For example, a systematic review showed that primary care and the community interventions, such as pedometers as motivational tools, motivational interviews, brief advice, GP prescription and GP counselling, appeared to be cost-effective, with ICERs ranging between €1161 and €16,666 per QALY gained [31, 67]. The ICER of €5206 per QALY gained we found in the current study compares very well with these estimates.

This study has a number of important strengths. It employed a previously used Markov model24,27 and extended it with a health state for depression. This allows for a broader assessment of the effects and costs associated with physical inactivity compared to previous studies. Moreover, a large number of alternative scenarios was evaluated using probabilistic sensitivity analyses, which allowed for an in-depth exploration of the uncertainty in assumptions and parameters used in the model. Finally, whenever possible, parameter estimates were selected from published meta-analyses to avoid the suggestion of “cherry-picking”.

Despite these strengths, the study also has some limitations. Although the most prevalent conditions associated with a lack of physical activity were included in the model, a number of other conditions have not been included (e.g. hypertension). We expect that this has led to an underestimation of the cost-effectiveness of the intervention rather than an overestimation. Also, it was not possible to have more than one disease at the same time in the model, whereas having for example diabetes increases the risk of cardiovascular disease as well. This was a pragmatic choice to keep the model as simple as possible. We expect that taking these comorbid risks into account could increase the potential cost-effectiveness of the intervention. In addition, there is a risk of double-counting the risk of mortality, as part of the mortality in the different physical activity states is probably associated with one of the five diseases included in the model. However, we expect that this effect is limited considering the relatively low risk of mortality for the different levels of physical activity compared to the mortality risks associated with the different health conditions included in the model. Finally, we assumed that the benefits of the intervention were sustained over the five-year period. Although empirical evidence on the sustainability of health behaviour changes is limited, studies indicate that interventions that target both physical activity and diet, are more likely to result in long-term changes in health behaviours [32, 68]. Moreover, results from the FFIT RCT, on which EuroFIT was based, showed that lifestyle changes were maintained over 3.5 years [18]. However, since we do not have reliable evidence on the retention of the changes in physical activity, we decided to limit the time horizon of the model to 5 years. When we assumed that effects disappear after 1 year, the ICER increased greatly (+ 553%). However, when we increased the time horizon to 10 years the ICER was considerably lower (− 233%).

Despite these limitations, this study is one of the first evaluating the longer-term cost-effectiveness of a novel lifestyle intervention that focussed on increasing physical activity and was tailored to men specifically. Further research should identify whether the EQ-5D-5 L is suitable to measure quality of life in relatively healthy men; data from the EuroFIT RCT suggests that its descriptive nature was not sensitive enough to distinguish between different levels of physical activity. Finally, longitudinal studies should show whether and to which extent effects on physical activity are sustained beyond the time horizon commonly employed in RCTs to better estimate long-term cost-effectiveness of interventions aimed at improving physical activity.

Based on the current study, we conclude the EuroFIT intervention not to be cost-effective compared to no intervention over a period of 5 years from a societal perspective. However, when using a time horizon of 10 years the results suggest that EuroFIT is more effective and less expensive compared to (i.e. dominant over) no intervention. We thus suggest that EuroFIT can potentially improve public health in a cost-effective manner in the long term.

## Supplementary information

**Additional file 1: Table S1**. Baseline characteristics of the population included in the EuroFIT trial reported as mean (SD) or N(%).

**Additional file 2:.**

## Data Availability

Data from the study are available for secondary analysis. Applications to access the data can be made by contacting the corresponding author of this paper.
