# Supplementary material for: Five-year cost-effectiveness analysis of the European Fans in Training (EuroFIT) physical activity intervention for men versus no intervention
Source: Int J Behav Nutr Phys Act. 2020 Mar 4;17:30. doi: 10.1186/s12966-020-00934-7 (PMC7055048; doi:10.1186/s12966-020-00934-7)
Supplement: Supplementary file 1 — Additional file 1: Table S1. Baseline characteristics of the population included in the EuroFIT trial reported as mean (SD) or N(%). [file 12966_2020_934_MOESM1_ESM.docx]

**Supplementary Table 1. Baseline characteristics of the population included in the EuroFIT trial reported as mean (SD) or N(%).**

|  |  | Intervention  (N=560) | Control  (N=553) |
| --- | --- | --- | --- |
| Age (years) | | 45.9 (9.0) | 45.6 (8.7) |
| Native to study country | | 501 (89.5%) | 482 (87.2%) |
| Years of Education | |  |  |
|  | <12 years  12-15 years  16+ years | 137 (24.5%)  205 (36.6%)  210 (37.5%) | 119 (21.5%)  216 (39.1%)  209 (37.8%) |
| Employment Status | |  |  |
|  | Working Full Time  Working Part Time  Not Working | 450 (81.7%)  32 (5.8%)  69 (12.3%) | 432 (79.6%)  43 (7.9%)  68 (12.3%) |
| Income^a^ | |  |  |
|  | Category 1 (Low)  Category 2  Category 3  Category 4  Category 5 (High)  Don’t know/Rather not answer | 36 (6.4%)  88 (15.7%)  113 (20.2%)  137 (24.5%)  127 (22.7%)  51 (9.1%) | 28 (5.1%)  100 (18.1%)  121 (21.9%)  132 (23.9%)  123 (22.2%)  40 (7.2%) |
| Relationship Status | |  |  |
|  | Married/Living with Partner | 439 (78.4%) | 447 (80.8%) |

^a^ Country-specific quintiles (low = lowest quintile of income in that country; high = highest quintile of income in that country)

SD = standard deviation
