# Supplementary material for: Five-year cost-effectiveness analysis of the European Fans in Training (EuroFIT) physical activity intervention for men versus no intervention
Source: Int J Behav Nutr Phys Act. 2020 Mar 4;17:30. doi: 10.1186/s12966-020-00934-7 (PMC7055048; doi:10.1186/s12966-020-00934-7)
Supplement: Supplementary file 2 — Additional file 2:. [file 12966_2020_934_MOESM2_ESM.docx]

| **Previous version of the EuroFIT model** | **Treatment arm** | **Total costs (€ 2017)** | **Total QALYs** | **Incremental Cost** | **Incremental QALYs** | **ICER** |
| --- | --- | --- | --- | --- | --- | --- |
| Base case | EuroFIT | €127,922,739 | 38,127 | €3,789,837  (1,074,047 ; 6,031,767) | 535  (509 ; 706) | 7,079 |
|  | No intervention | €124,132,902 | 37,592 |  |  |  |
| Healthcare perspective | EuroFIT | €49,805,648 | 38,127 | €3,050,619  (1, 844,907 ; 4,178,542) | 535  (508 ; 704) | 5,698 |
|  | No intervention | €46,755,029 | 37,592 |  |  |  |
| Utility values from the literature | EuroFIT | €125,195,147 | 38,163 | €2,371,709  (1,294,099 ; 3,571,741) | 244  (246 ; 309) | 9,703 |
|  | No intervention | €122,823,438 | 37,919 |  |  |  |
| EuroFIT effectiveness lasts only one year | EuroFIT | €123,045,962 | 36,595 | €3,862,362  (3,115,307 ; 4,727,796) | 504  (507 ; 651) | 7,662 |
|  | No intervention | €119,183,600 | 36,091 |  |  |  |
| Time horizon 10 years | EuroFIT | €200,499,449 | 60,434 | €6,237,349  (1,481,122 ; 10,284,491 ) | 1,505  (1,356 ;2,044) | 4,145 |
|  | No intervention | €194,262,100 | 58,930 |  |  |  |
| **Current version of the EuroFIT model** | **Treatment arm** | **Total costs (€ 2017)** | **Total QALYs** | **Incremental Cost** | **Incremental QALYs** | **ICER** |
| Base case | EuroFIT | €146,629,613 | 40,531 | €654,611  (-73,893,166 ; 81,741,624) | 126  (-1999 ; 2527) | 5206 |
|  | No intervention | €145,975,002 | 40,405 |  |  |  |
| Healthcare perspective | EuroFIT | €72,489,139 | 40,531 | €496,731  (-37,026,528 ; 35,107,767) | 126  (-2026 ; 2537) | 3951 |
|  | No intervention | €71,992,408 | 40,405 |  |  |  |
| Utility values from the literature | EuroFIT | €146,629,613 | 39,767 | €654,611  (-73,893,166 ; 81,741,624) | 564  (-68 ; 1106) | 1161 |
|  | No intervention | €145,975,002 | 39,203 |  |  |  |
| EuroFIT effectiveness lasts only one year | EuroFIT | €147,631,100 | 40,427 | €1,759,289  (-19,869,112 ; 25,189,547) | 52  (-555 ; 725) | 33,997 |
|  | No intervention | €145,871,811 | 40,375 |  |  |  |
| Time horizon 10 years | EuroFIT | €303,622,814 | 71010 | -€2,595,287  (-128,814,119 ; 137,269,728) | 373  (-3254 ; 4534) | -6949 |
|  | No intervention | €306,218,101 | 70636 |  |  |  |
